# Modeling Behavioral Response to Vaccination Using Public Goods Game

**DOI:** 10.1109/TCSS.2019.2896227

**Published:** 2019-03-07

**Authors:** Marzieh Soltanolkottabi, David Ben-Arieh, Chih-Hang Wu

**Affiliations:** Department of Industrial and Manufacturing Systems EngineeringKansas State University 5308 ManhattanKS66506USA

**Keywords:** Disease transmission, public goods game, vaccination

## Abstract

Epidemics of infectious disease can be traced back to the early days of mankind. Only in the last two centuries vaccination has become a viable strategy to prevent such epidemics. In addition to the clinical efficacy of this strategy, the behavior and public attitudes affect the success of vaccines. This paper describes modeling the efficacy of vaccination considering the cost and benefit of vaccination to individual players. The model is based on the public goods game and is presented as a spatial game on a lattice. Using this model, individuals can contribute to the public health by paying the cost of vaccination or choose to be protected by the public who is vaccinated rather than pay the cost and share the risk of vaccination. Thus, in this model individuals can choose to stay susceptible, can become infected, or choose to vaccinate once in each episode. This paper presents the behavioral changes of the population and the cost to the society as a function of the cost of vaccines, cost of being infected, and the “fear factor” created by the public media.

## I. Introduction

Epidemics of infectious disease have influenced the human civilization for many centuries. Epidemics have been documented as early as 430-427 BCE when the Athens epidemic killed as much as half of the population of ancient Athens [1]. From early times, man has tried to understand the causes and remedies to infectious diseases, epidemics that took a huge toll on the human civilization. Only after the later part of the nineteenth century did vaccination become a successful tool in the fight against infectious disease.

In recent decades, the shift in climate and global temperature has raised the concern of increasing exposure to human’s infectious diseases [2]. The World Health Organization (WHO) has stated that the climate change and warming of the atmosphere are likely the causes of increase in transmission of many infectious diseases [3]. Similarly, the *New England Journal of Medicine* has raised the concern that the climate change will cause a significant increase in infectious disease, especially vector-borne and waterborne diseases [4]. This is due to the fact that insect vectors tend to be more active at higher temperatures. Moreover, there are also suspicions that the climate change has caused certain infectious diseases to spread into geographical areas that were previously unaffected [5].

Another concern raised in a report conducted by WHO is that globalization and its effect on economic, environmental, demographic, and topological change of societies has caused people in today’s world to be in an increased risk of confronting infectious disease [6].

All these factors reemphasize the importance of controlling the spread of infectious diseases and motivated new approaches toward modeling infectious disease transmission and the behavioral response to it.

Many studies have considered social and behavioral dynamics in impeding the spread of infectious disease since these behaviors can influence the dynamics of the spread of disease in populations [7], [8]. This behavioral or health belief model has traditionally considered four main factors [9]: 1) the perceived susceptibility of an individual or the probability that a person become infected; 2) the perceived severity or the cost of being infected; 3) the perceived barriers to behavior adoption or the cost of prevention; and 4) the perceived benefits or the benefits of adopting a behavior.

**Fig. 1.**
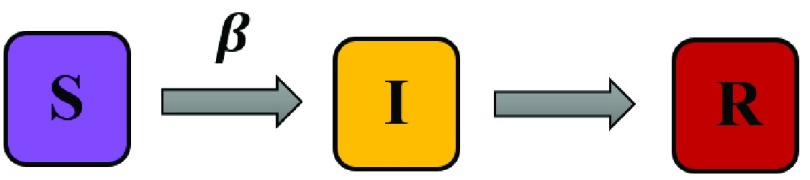
Schematic illustration of the SIR model. The parameter $\beta $ denotes the transmission rate.

Moreover, in the classical modeling of infectious disease spread, a population is typically classified into several categories based on the individual’s disease status. These categories are susceptible individuals (S), infected individuals (I), and recovered individuals which become immune. This model is known as the SIR model and is wildly used in studying infectious disease transmission [8]. Fig. 1 shows the schematic illustration of the SIR model, in which the susceptible individuals can become infected by the transmission rate and per contact probability of $\beta $, and every infected individual will recover after some time steps ($L$).

**Fig. 2.**
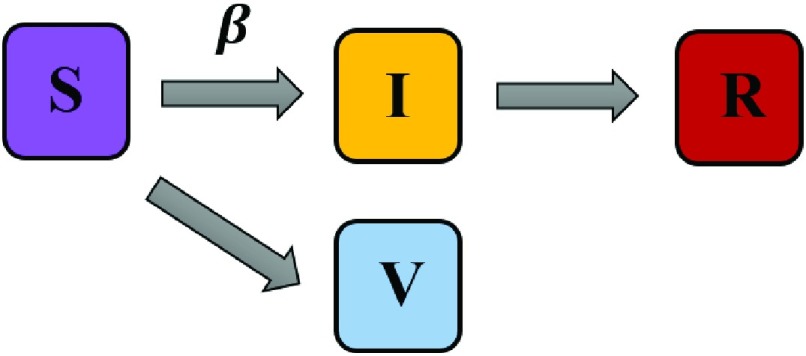
Schematic illustration of the SIRV model. The parameter $\beta $ denotes the transmission rate.

When considering a behavioral response to the epidemic such as vaccination, the SIR model adds one more state to the individuals who have used the prevention technique and the model is upgraded to the following form referred to as SIRV model (V for vaccinated) as shown in Fig. 2. In this model, states R and V are the absorbing states.

The spread of infectious disease can be studied at the population level or based on a social interacting network. The network-based model can better describe the spread of infectious diseases since many diseases are transmitted with a direct or close contact between individuals. The network-based models have been widely studied in recent research works. For example, Fu *et al.* [10] modeled the role of imitation behavior in a spatial structure and showed that the spatial structure is more sensitive to changes in the cost of vaccination. Fukuda *et al.* [11] applied the same model but instead of one network of interactions, they defined two networks, in which one is the disease transmission network and the other one is the information transmission network.

In spite of vaccination being a proven and potent remedy to the spread of infectious diseases, in some regions of the U.S., there is a growing trend of parents opting out of vaccinating their children [12]. Moreover, vaccines are not required in order for children to attend school in most states. Some national organizations such as “Vaccination Liberation” actively promote exemptions and encouraging physicians to refuse to vaccinate [13].

When studying the reasons that deter parents from vaccinating their children, the most common reasons are fear from side effects or harm to the child [12]. It seems that counting on the other vaccinated children to provide protection is not among the leading reasons. However, as a matter of fact, this behavior exemplifies the “tragedy of the commons” as the behavior that these parents are choosing to subscribe to.

In order to study the behavior of individuals in response to epidemic disease, several methods have been used. The evolutionary game theory is one of the techniques that can adequately describe the choice of individuals in population of interacting players with different payoffs [14]. While prevention of infectious diseases is usually achieved by increasing the number of individuals who are using prevention techniques such as vaccinating or social distances, some individuals will choose not to get vaccinated being “protected” by the vaccinated population. The idea of being protected by the population of vaccinated individuals is not always effective if there are not enough vaccinated individuals or motivators to encourage individuals to get vaccinated. To illustrate, in 2015, California faced the worst Measles epidemic in the last decades because many parents declined to vaccinate their children due to vaccine side effects and by blindly relying on the other vaccinated ones to be safe [15]. However, the idea of free protection has always been partnered with voluntary vaccination.

Considering this “free protection” allows modeling the spread of an infectious disease as a public goods games in which some free riders get the advantage from the contribution of other individuals in the group (i.e., vaccination). Modeling a public goods game of infection versus vaccination uses a payoff function, in which a vaccinated individual has a cost equal to cost of vaccination, an infected one pays the cost of infection, and the free riders are the ones deciding not to be vaccinated and when do not get infected pay nothing [10], [11], [16].

In this paper, we have developed a public goods game-based model, in which the payoff of each individual is calculated based on its share of perceived cost of susceptibility, severity, and barriers for the whole group where he is a member, rather than his personal cost of vaccination or infection. The spatial game theory has been used to consider the spatial network-based structure of the population of players. In this model, in every time step, all susceptible players update their strategy for getting vaccine or not synchronously and then each of them might be infected based on its strategy and the probability of becoming infected. In order to update the strategy to get vaccine or not, different methods have been used. In the classical method of updating strategy, each player updates its strategy to the strategy of the player with the highest payoff in its neighborhood [14]. In many other studies, the Fermi function is used as a probability function to change to the strategy of a neighbor [10], [16]–[18]. In this paper, a new approach in updating the strategy is used in which each susceptible player updates its strategy not just to the strategy of the neighbor with the highest payoff but also to the strategy of the number of neighbors with the highest payoffs. Thus, if there is someone vaccinated among the neighbors with the highest payoff, the player will change its strategy to get the vaccine. The sensitivity factor is a measure for the number of neighbors with the highest payoff that a player refers to in order to update its strategy. This sensitivity factor can be considered as a surrogate form of fear factor or the effect of media on making people aware of the severity of a disease. The concept of the effect of media and fear factor on individual’s decision is studied in some research trying to show how it can affect the spread of infectious diseases [19]–[22].

In Section II, the public goods game and the proposed methodology in modeling spread of infectious disease is presented. Section III presents the result of using this methodology considering different parameters and discussing the behavior of individuals facing infectious disease outbreaks. Section IV provides a summary, discussion, and future work.

## II. Methodology

In this paper, individuals’ cost functions and payoffs are determined based on their contribution to the group and the group’s shared payoff which can be seen as a public goods game. This means that any payoff in a group will be distributed among the members of the group. The payoff function for a typical public goods game is as follows [23]:

\begin{align*} {\mathrm {payoff}}_{i}=\begin{cases} \dfrac {rN_{c}C}{N}, &\mathrm {if}~i~\mathrm {is~not~cooperative} \\[0.5pc] \dfrac {rN_{c}C}{N}-C, &\mathrm {if}~i~\mathrm {is~cooperative} \end{cases}\tag{1}\end{align*}

In which $N_{c}$ is the number of cooperative individuals, $C$ is the cost of cooperation (or contribution to the public welfare), $N$ is the total number of members in a group, and $r$ is the multiplication factor.

**Fig. 3.**
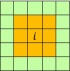
Cooperation group for individual $i$.

In our model, we have considered a person and all its immediate neighbors as a group; so, in a lattice, the center cell is the player in question, and its cooperation group is the eight cells adjacent to it, as shown in Fig. 3, where the yellow cells show the cooperation group for player $i$.

The payoff for being a member of this group is defined as follows:

\begin{align*}&\hspace {-0.5pc}{\mathrm {Payoff}}_{g}=-\left ({\left ({\frac {N_{I}}{N}\mathrm {\times }C_{I} }\right)+\left ({\frac {N_{V}}{N}\times C_{V} }\right)}\right. \\&\qquad\qquad\qquad{{\displaystyle {+\,\left.{+\left ({\frac {C_{I}\times \sum \limits _{j\mathrm {\in }S} P_{\inf j}}{N} }\right)\left ({\frac {N_{R}}{N}\mathrm {\times }C_{R} }\right) }\right)} }}\tag{2}\end{align*}

where $N_{I}$ is the number of infected individuals in a group, $C_{I}$ is the cost of infection, $N_{V}$ is the number of vaccinated individuals in a group, $C_{V}$ is the cost of vaccination, $S$ is the group of susceptible members, $P_{\inf j}$ is the probability of getting infected for player $j$, $N_{R}$ is the number of recovered individuals in a group, $C_{R}$ is the cost of being recovered for the group, and $N$ is the total number of members of a group. In other words, we can say that the first and last portions of the equation show the perceived severity, the second portion is the perceived barriers, and the third portion is the perceived susceptibility of a group. Note that the payoff is negative, treated as a cost rather than a benefit.

Also, in this function, $P_{\inf j}$ can be calculated using the following formula [24]:

\begin{equation*} P_{\inf j}=\frac {N_{Ij}}{N}\times \beta\tag{3}\end{equation*}

where $N_{I,j}$ is the number of infected neighbors of $j$, $N$ is the total number of neighbors of $j$ and $\beta $ is the disease’s transmission rate based on a one-on-one contact.

The total payoff of a player is equal to the payoff that a player can earn from participating in a group minus the cost of being infected or getting the vaccine. The following formula shows the total payoff of a player:

\begin{align*} {\mathrm {Payoff}}_{i}=\begin{cases} {\mathrm {Payoff}}_{g}-C_{V},&\mathrm {if}~i~\mathrm {is~vaccinated} \\ {\mathrm {Payoff}}_{g}-C_{I},&\text {if}~i~\mathrm {is~infected~or~recovered} \\ {\mathrm {Payoff}}_{g},&\mathrm {if}~i~\mathrm {is~susceptible~(free~rider)}. \end{cases} \tag{4}\end{align*}

### A. Updating Rule

The updating rule is such that in every time step the top $s$ neighbors with the highest payoff in the neighborhood of a player will be chosen, and if there is someone vaccinated among them and its payoff is higher than the payoff of that player itself, the player will decide to get vaccinated; otherwise, it remains susceptible. We call $s$ the sensitivity factor.

**Fig. 4.**
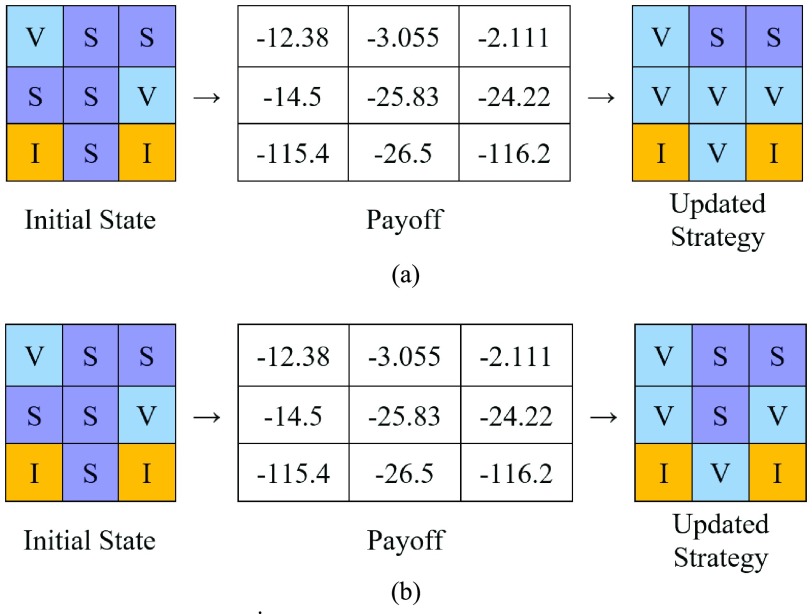
Illustration of the updating strategy. (a) S = 3. (b) S = 2.

To illustrate, in the following lattice, consider the sensitivity factor equal to 3. The set of the first three neighbors with the highest payoff will be $\{(-2.111,S), (-3.055,S)$, and $(-12.38,V)\}$ (Fig. 4). The updating strategy of the center player will be to vaccinate if one of the top three neighbors is vaccinated, which is the case in Fig. 4(a). If we change the sensitivity factor to 2, then the player will not vaccinate, as shown in Fig. 4(b), since the top two players are not vaccinated. The numbers in the cells show the payoff for the center player and its neighbors, and the payoff of neighbors is calculated based on their eight neighbors, some of which are not shown here. We have considered $(C_{I}, C_{V},C_{R},\beta)$ equal to (100, 10, 0, 0.2).

## III. Experimental Results

In the following, the results of changing different factors that can affect the dynamics of an epidemic are studied. In the lattices, purple cells show susceptible, yellow cells show infected, red cells show recovered, and light blue cells show vaccinated individuals. The parameters which are used in the models are presented in Table I.

**TABLE I:** Parameters of the Model

Parameter	Meaning	Value
$N$	Population size	2500
$I_{0}$	Percentage of initially infected individuals	5%
$R_{0}$	Percentage of initially recovered individuals	0%
$L$	Duration of infectious period	19 time steps
$C_{R}$	Cost of being recovered	0
$C_{I}$	Cost of infection	1000
$C_{V}$	Cost of vaccination	Variable
$\beta $	Transmission rate	Variable
$V_{0}$	Percentage of initially vaccinated individuals	Variable
$s$	Sensitivity factor	Variable

### A. Effect of Changing Cost of Vaccination

One of the variables that can affect the spread of infectious disease and is under control of the health policy makers is the cost of vaccination. It is reasonable for the cost of vaccination to be less than the cost of infection and practically it should be much less than the cost of infection otherwise people will prefer not to pay the cost of vaccination. Thus, to examine the effect of $C_{V}$ on the epidemic and the number of vaccinated individuals, a simulation model was generated. The model was run on a $50\times50$ lattice, in which the initial number of infected and vaccinated individuals is distributed randomly. It is updated for 200 time steps to make sure that epidemic has reached the steady-state situation, and then the result of the steady-state lattice is used for evaluations.

**Fig. 5.**
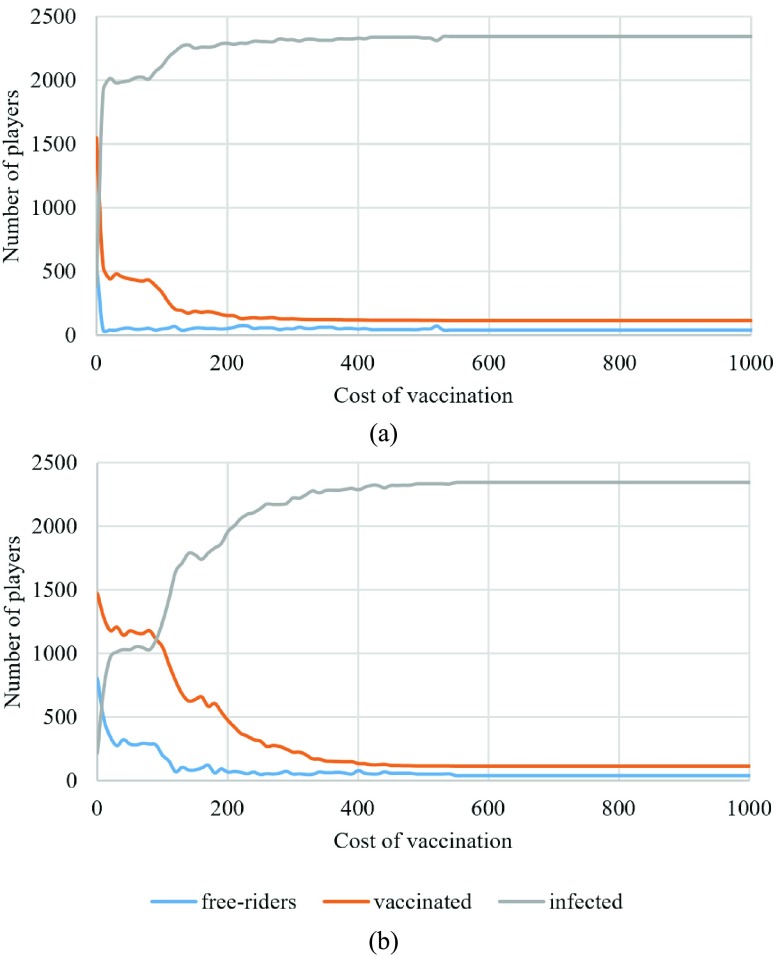
Number of free riders, infected, and vaccinated individuals for different vaccination costs.

In Fig. 5, the results of updating a lattice using different vaccination costs from 0 to a cost equal to the cost of infection is presented.

**Fig. 6.**
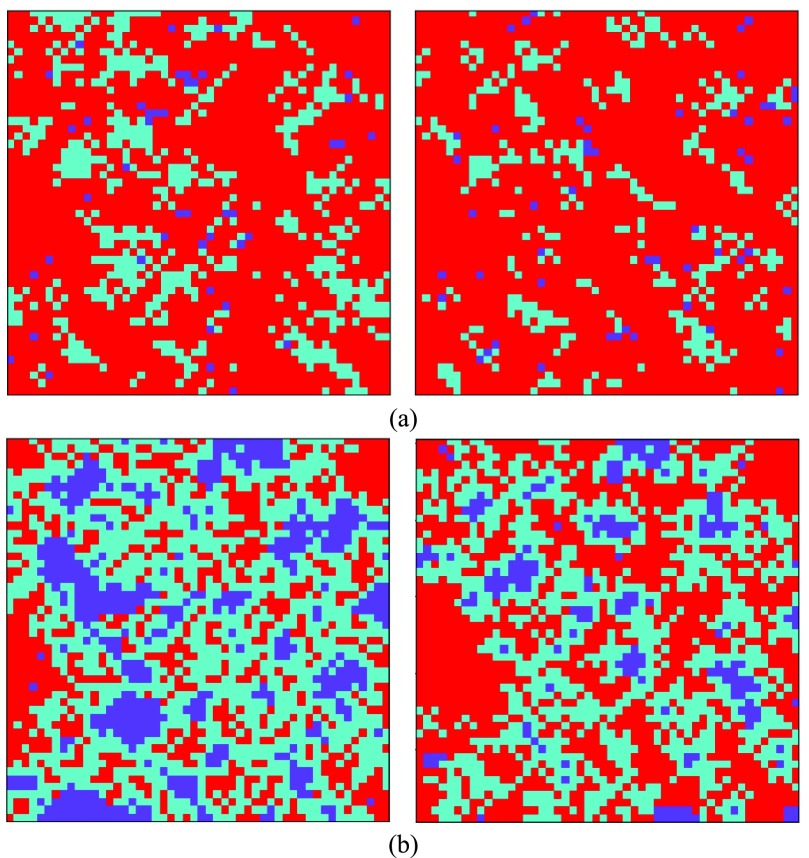
Distribution of players in the last lattice for $C_{V}=10 \mathrm {and} 100$ when (a) $s=1$ and (b) $s=4$.

Fig. 5(a) shows the number of free riders, infected, and vaccinated individuals under different vaccination costs when $s=1$. Fig. 5(a) shows that as predicted increasing the vaccination cost will decrease the number of vaccination to the point that no one decides to get vaccinated other than the initially vaccinated ones. However, comparing this result with the one with a higher sensitivity factor [$s=4$, Fig. 5(b)] shows that increasing the sensitivity can result in more vaccinated individuals and also more free riders which is beneficial to the society. Moreover, in the experiments, it can be seen that higher sensitivity factor can result in better control of the epidemic for any vaccination cost $C_{V}$ as long as the value of $C_{V}$ is not too high resulting in no additional vaccinated individual. These phenomena can be seen from Fig. 6(a) and (b). Fig. 6(a) shows the result of updating the lattice when $C_{V}=10$ (left) and $C_{V}=100$ (right) for $s=1$, and Fig. 6(b) shows the result of updating the lattice when $C_{V}=10$ (left) and $C_{V}=100$ (right) for $s=4$. It can be seen that when $s$ is higher there are some clusters of vaccinated individuals who surround infected individuals and cause the epidemic to be controlled more effectively. As a result, the number of free riders (purple cells) increases because there is enough protection provided by vaccinated individuals. Thus, the society benefits from a lower cost of vaccination and disease in comparison with the same level of protection when all the individuals are vaccinated. It is worth to mention that the epidemic season length did not show a meaningful relation with the cost of vaccination.

This also shows the benefit of increasing $s$, indicating a higher public awareness of the risk and harm of the disease. Increasing $s$ can be done practically by using public media, social networks, and similar mass communication channels.

### B. Effect of Changing Transmission Rate

**Fig. 7.**
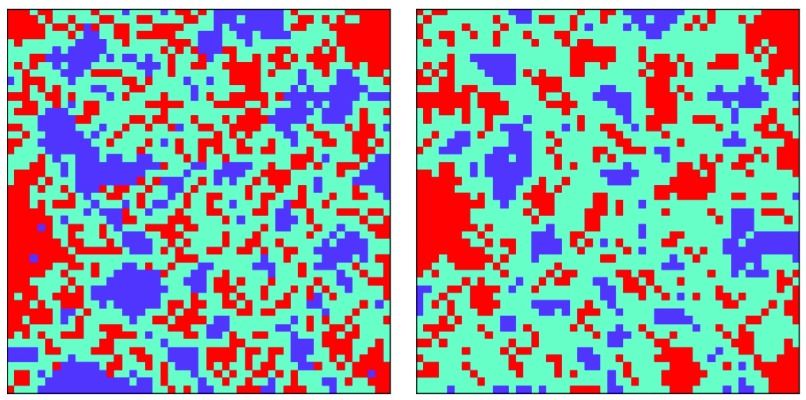
Distribution of players in the last lattice when $\beta =0.2$ (left) and $\beta =0.9$ (right).

**Fig. 8.**
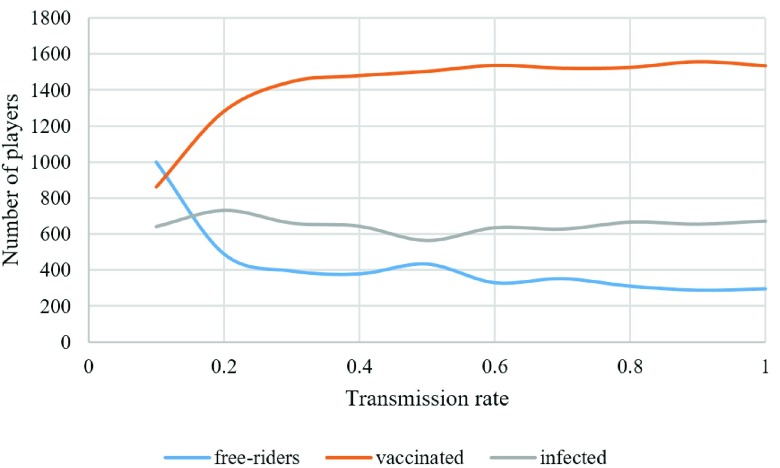
Number of vaccinated, infected, and free riders for different transmission rates.

The disease transmission rate is another variable in the model which depends on the characteristic of the disease. In order to examine the behavior of this model facing different diseases with different transmission rates, the number of finally vaccinated and infected individuals for different transmission rates is studied. In Fig. 7, the lattice on the left side shows the result of updating a $50\times50$ lattice when $C_{V}=10$ and $s=4$ for $\beta =0.2$, and the lattice on the right side shows the result of updating a $50\times50$ lattice when $C_{V}=10$ and $s=4$ for $\beta =0.9$. It can be seen that more people get vaccinated when the transmission rate is high (especially in the lower rates of transmission) to save themselves and their community. This phenomenon can be better seen in Fig. 8, in which the number of vaccinated, infected, and free riders is plotted for different transmission rates from 0.1 to 1. Fig. 8 shows that the public behavior is sensitive to the transmission rate at the lower end and is more stable at high rates of transmission. This sensitivity was experienced during the 2003 SARS episode in Hong Kong [9].

**Fig. 9.**
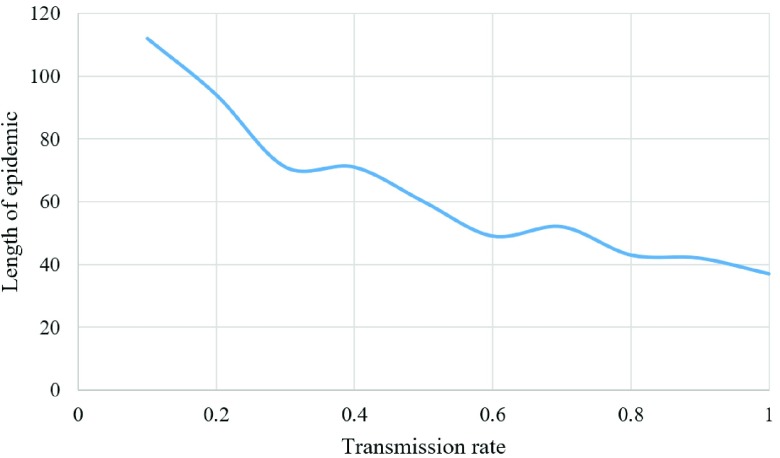
Length of epidemic for different transmission rates.

Moreover, the epidemic tends to end sooner with a higher transmission rate, due to the faster response of individuals to the epidemic because of its high threat to the players (i.e., higher cost) as shown in Fig. 9.

### C. Effect of Changing Initially Vaccinated Population

**Fig. 10.**
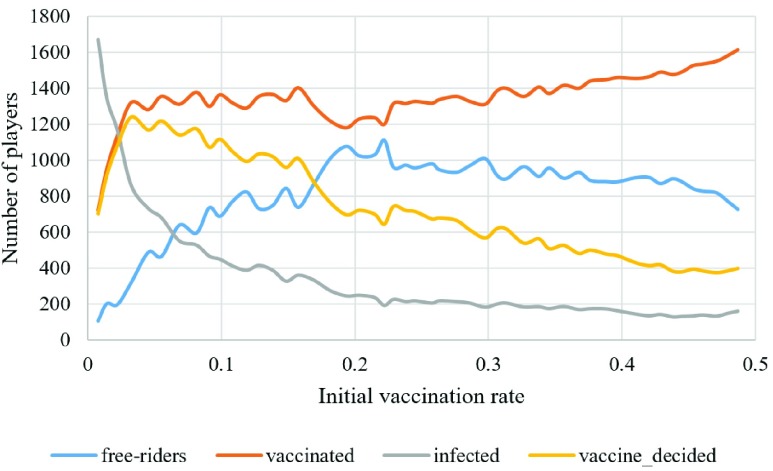
Number of vaccinated, infected, and free riders for different initial vaccination rates.

The number of initially vaccinated individuals in the population is another parameter that can be controlled by the health policy makers by forcing some individuals in the population to get vaccinated either using free-subsidy policy or partial-subsidy policy. The result of changing the percentage of initially vaccinated individuals shows that increasing the percentage of vaccinated individuals is beneficial to the society as long as the number of vaccinated is not beyond a certain percentage causing some individuals to get the benefits of living among vaccinated individuals in an immune society. As it is apparently shown in Fig. 10, the number of free riders will increase as the number of initially vaccinated increase, but it will start to decrease as the number of vaccinated increase more than 20%. These experiments are done in a $50\,\times \,50$ lattice when $C_{V}=10$, $\beta =0.2$, and $s=4$, while changing the percentage of initially vaccinated ones ($V_{0}$) from 1% to 50%. Moreover, the number of finally vaccinated individuals does not exhibit a large change for different experiments while as the number of initially vaccinated ones increases the number of individuals who decide to get vaccine decrease. This can be better seen when we plot the number of individuals who decide to get vaccine during the epidemic (Fig. 10 —yellow line). This decrease in the number of voluntary vaccination can be explained by the “group protection” that more vaccinated individuals provide. In addition, the graph of infected individuals shows that as we increase the number of initially vaccinated people linearly, the number of infected people will decrease much faster. It is worth mentioning that in all the experiments for Fig. 10, the number of initially infected individuals was 5% of the population.

**Fig. 11.**
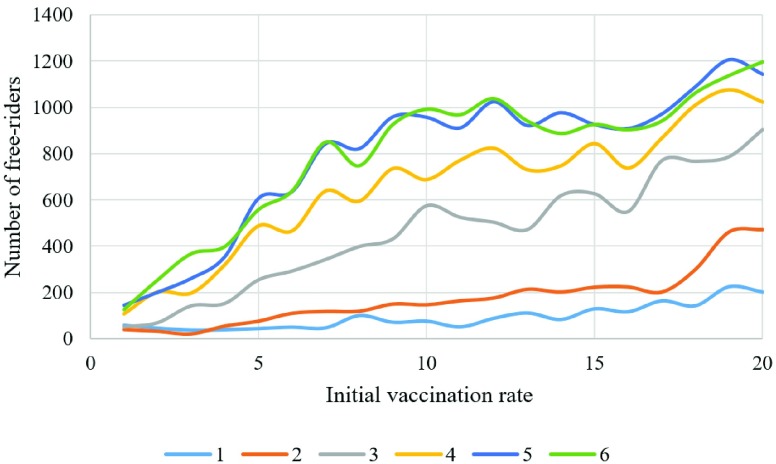
Number free riders for different initial vaccination rates and sensitivity factors.

Studying the behavior of population using different sensitivity factors and initial vaccination rate shows that the higher sensitivity will cause the number of free riders to grow faster. In Fig. 11, each line shows the number of free riders for each value of $s$ from 1 to 6, and it is apparent that as the value of $s$ increases the number of free riders increase. This can be explained by the fact that increasing the number of vaccinated individuals in the cooperation group provides more protection and reduces the number of infected individuals and reduces the need of individuals to get vaccinated. It is worth to add that this result is true when the number of initially vaccinated is less than 20% because the number of free riders will start to decrease as we increase the number of initially vaccinated individuals as it can be seen from Fig. 10.

### D. Effect of Changing Initial Distribution of Players in the Lattice

**Fig. 12.**
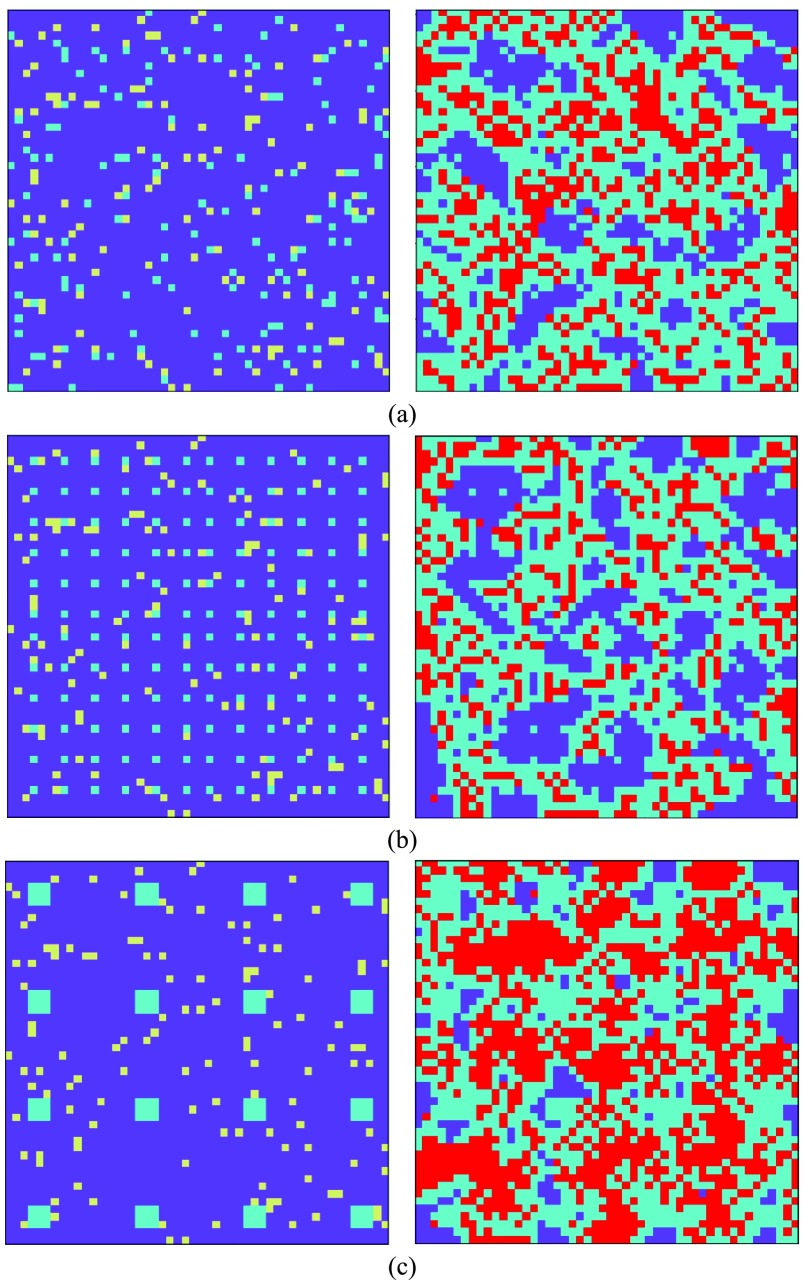
First and last lattices for different distributions of vaccinated individuals.

The distribution of players in the lattice is also a factor that can be controlled to achieve better vaccine coverage. Although the health policy makers have control over the initially vaccinated individuals in society, they have no control over the number of initially infected ones. Thus, to model the effect of changing distribution of players, the initially infected individuals are randomly distributed in the lattice, but three scenarios are considered. In the first scenario, the vaccinated individuals are distributed randomly in the population [Fig. 12(a)], in the second one, the vaccinated individuals are evenly distributed in the lattice [Fig. 12(b)] and in the third scenario, the vaccinated individuals grouped into larger clusters (of nine individuals) that are evenly distributed in the lattice [Fig. 12(c)]. In all scenarios, the number of vaccinated and infected individuals is the same. These experiments are done in a $50\times50$ lattice considering $C_{V}=10$, $\beta =0.2$, and $s=4$.

Table II shows the result of each scenario. In all the three scenarios, the number of initially vaccinated is 144 which is approximately 5% of the population. We can see from Table II that if the individuals are evenly distributed, we will have few numbers of infection and vaccination and more free riders. This result is the effect of accessibility of vaccinated individuals in all cooperation groups; so, individuals can decide sooner to get vaccine confronting a disease epidemic and can also save others from being infected. Moreover, the result shows that group vaccination is less effective while there might be some vaccination not necessary for people who are not at risk of being infected, and also more initial vaccinations are needed to support this society. However, if we distribute groups such that they are more reachable for other individuals for imitating their behavior, the result will be improved.

**TABLE II:** Parameters of the Model

Scenario	Free-Riders	Vaccinated	Infected	Length of Epidemic
1 (a)	517	1347	636	98
2 (b)	759	1240	501	90
3 (c)	232	1205	1063	100

### E. Effect of Sensitivity Factor

**Fig. 13.**
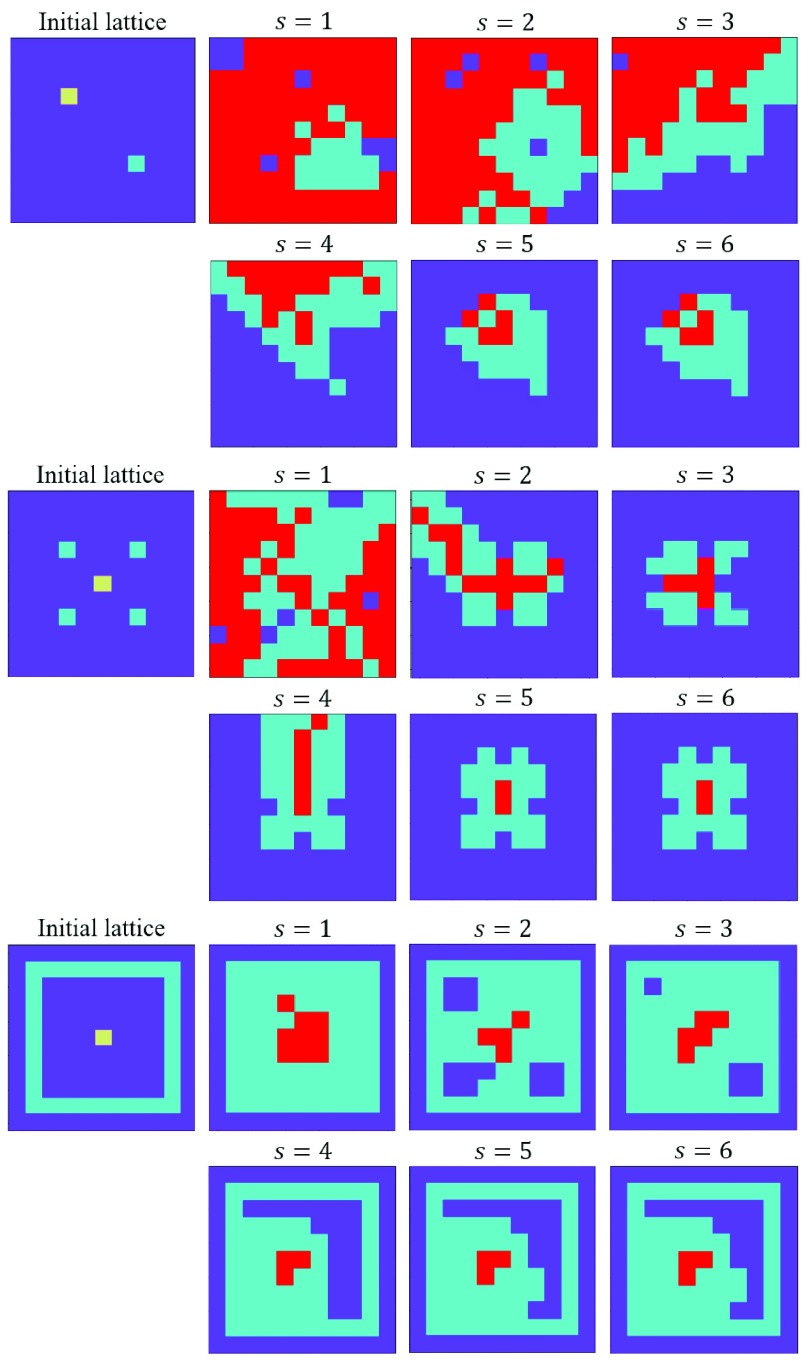
Results of updating three custom starting lattices.

In order to examine the behavior of the model to changing sensitivity factor, the steady-state results of updating three different starting lattices under different sensitivity factors are presented in Fig. 13. Fig. 13 shows that as the sensitivity factor increases the infection has a lower chance to affect other people while individuals respond sooner to epidemic. In these experiments, we have considered $C_{V}=1$ and $\beta =0.2$. It can be seen in Fig. 13 that as sensitivity factor increases in all three cases, vaccination has a better coverage and vaccinated individuals can better control the spread of an infectious disease.

**Fig. 14.**
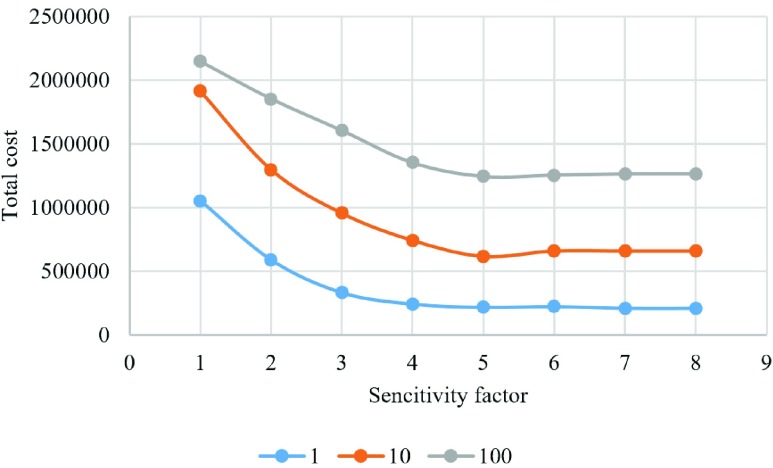
Total cost of each experiment.

**Fig. 15.**
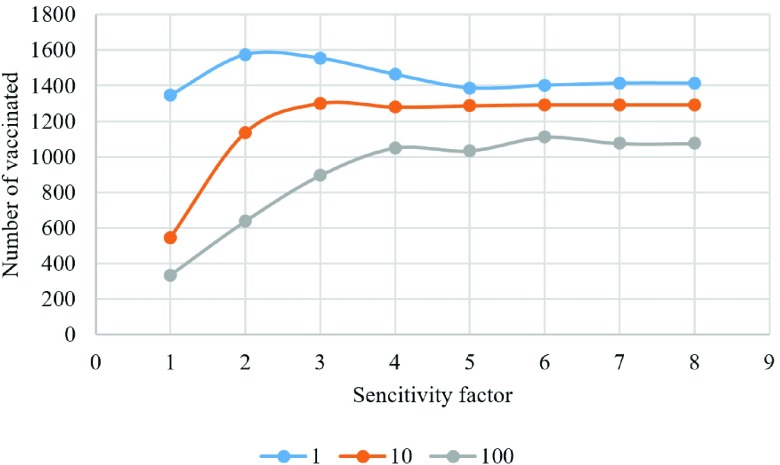
Number of vaccinated individuals in each experiment.

**Fig. 16.**
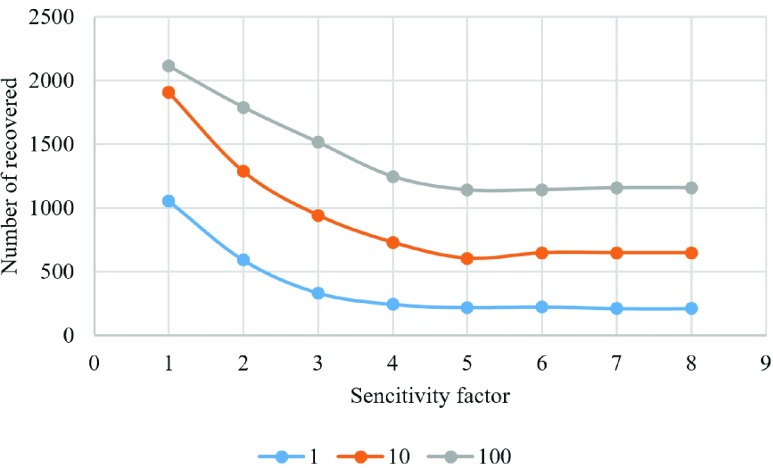
Number of recovered individuals in each experiment.

**Fig. 17.**
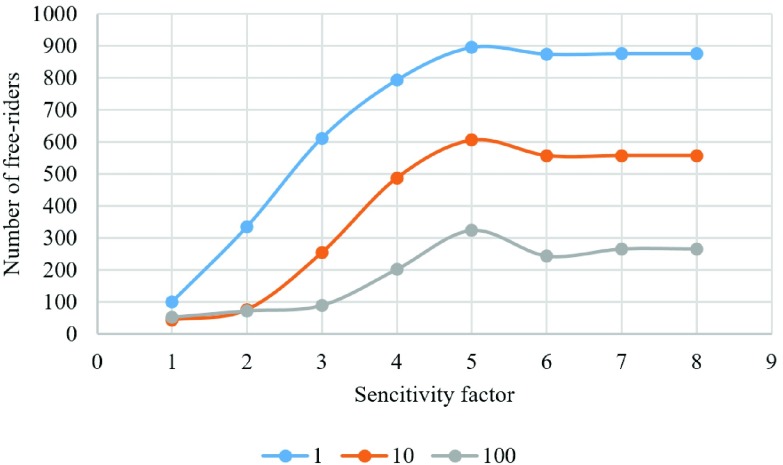
Number of free riders in each experiment.

**Fig. 18.**
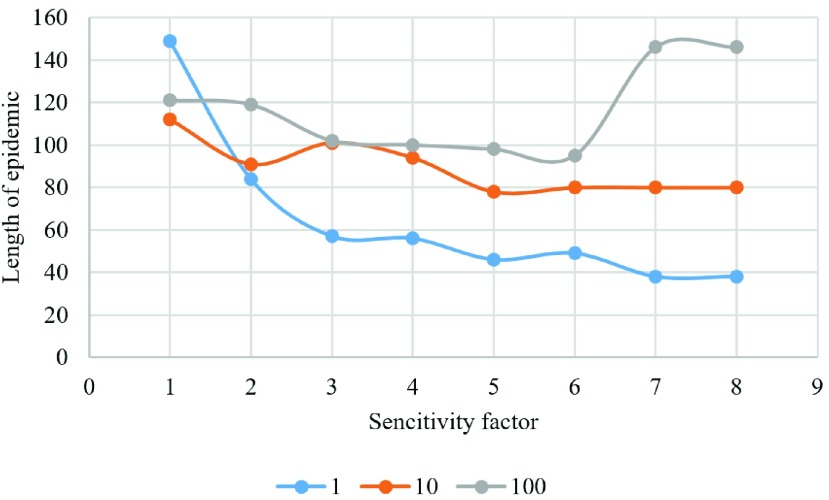
Length of epidemic season in each experiment.

We have also examined the result of changing sensitivity factor when updating a lattice with distribution of players for three different vaccination costs (Figs. 14–18). In the diagrams, the blue lines show the experiments for $C_{v}=1$, orange lines show the experiments for $C_{v}=10$, and gray lines show the experiments for $C_{v}=100$.

Fig. 14 illustrates the total cost of each lattice of players. This cost is calculated using the following formula which shows the total cost of being infected or vaccinated for the society:

\begin{equation*} \mathrm {Total cost=}C_{I}({N}_{I}+N_{R}){+C}_{V}N_{V}.\tag{5}\end{equation*}

In this case, $N_{I}$, $N_{R}$, and $N_{V}$ are the number of infected, recovered, and vaccinated individuals in the entire lattice.

We can see that as the number of free riders increases, and the ratio of infected-to-vaccinated individuals decreases, the total cost decreases. This means that the society could control the spread of disease with minimum number of vaccinated individuals. In Fig. 14, the total cost decreases for all three different vaccination costs as the sensitivity factor increases but it does not change much for $s\ge 5$.

Fig. 15 shows the number of vaccinated players in the last lattice for each scenario. We can see that for sensitivity factors greater than 4, the number of vaccinated individuals does not change notably. However, the final number of vaccinated individuals is greater when the cost of vaccination is low.

Fig. 16 shows the number of recovered individuals in the last lattice. In the last lattice, the epidemic season is over, and all the infected individuals have changed their state to “recovered.” Thus, the number of recovered individuals is used to represent the number of individuals infected during each run. Fig. 16 shows that the number of recovered individuals has the same behavior as the total cost; this is due to the high cost of infection relative to the cost of vaccination and the effect of the high number of infected individuals on the total cost. Also, Fig. 16 demonstrates again that more people will be infected as the cost of vaccine increases.

Fig. 17 shows the number of free riders in each experiment. As can be seen, this number increases as sensitivity increases but levels off after $s=5$. Based on this output, we conclude that the sensitivity factor that maximizes the number of free riders is $s=5$ independently of the cost of vaccination. It is interesting to observe as discussed earlier that a higher $s$ factor that relates to the fear of individuals from being infected is proportional to the number of free riders (who choose not to vaccinate). This counter intuitive result can be explained as a society that is more active in protecting itself also provides protection to free riders who benefit from this anxiety. This shows that the best policy in society is to follow the majority of the comparison group.

Fig. 18 shows the duration of the epidemic season in each experiment. Based on this result, we can conclude that the length of an episode is mainly affected by the cost of vaccination; since cheaper vaccination results in an increase in the number of vaccinated individuals and cause the epidemic to end sooner. Similarly, if the sensitivity factor is increased, the duration of an epidemic is reduced. This is explained by the society that exhibits a higher sensitivity to the risk of being infected and is more proactive in vaccinating.

## IV. Conclusion

In this paper, a public goods game-based model for modeling the behavior of population of players in response to an epidemic is illustrated. In this model, the payoff of each player is calculated using a function in which every cost of individuals in a $3\times 3$ group is divided between the members of that group. These costs are the cost of infection for infected people in the group, cost of vaccination for vaccinated individuals, probable cost of being infected for susceptible people, and cost of being recovered for recovered individuals. Using the payoff of each player, individuals try to imitate the behavior of the people who are in the groups with the lowest cost or the highest payoff. The sensitivity factor is one of the parameters which is introduced to show the risk tolerance (fear) of players encouraging switching to the strategy of their neighbors. The sensitivity factor can show the number of neighbors with the highest payoffs in the candidate list of the players; so, if any vaccinated individual is in this candidate list, the player will be encouraged to get vaccinated. Although in our model, the sensitivity factor is not varying among players and is considered to be influenced by the social media, it can cause different behaviors in populations. Using this model, we can show that if the cost of vaccination is increased, players have less tendency to get vaccinated, which is a representative behavior to a real-world situation.

However, increasing the sensitivity of individuals can result in more vaccination in the same situation. This behavior is very similar to the effect of fear of being infected in the real-world epidemics. Moreover, increasing the sensitivity can be beneficial for the society as individuals react to the epidemic sooner and decide faster to get vaccine in order to save themselves and their community, but increasing the sensitivity factor too much does not lead to an optimal cost for the society. The results show that increasing the sensitivity factor to more than 5 does reduce cost while the number of free riders dose not increase and the number of vaccinated and infected individuals does not change. This behavior is the result of dissuasive effect of selecting the strategy of the neighbors who have a higher payoff than the payoff of the player itself on the candidate list.

In addition, the model tests the effect of the infection transmission rate, and surprisingly, the epidemic length is lower when facing a disease with high transmission rate. This is explained by the fact that individuals respond sooner to the disease spread when there is a higher probability of being infected (represented as a strategy with a higher potential cost).

In this model, we also examined the effect of the number of initially vaccinated individuals on the epidemic which shows that mandatory vaccination can be beneficial when it does not force too many individuals to get the vaccine. Also, the distribution of vaccinated players in the lattice can affect the final result, when the players are distributed evenly in the lattice, more people are in contact with vaccinated individuals and this can cause them to get vaccine sooner when facing an epidemic and can result a better control of epidemic compared to the same number of vaccinated individuals who are randomly distributed.
